# Improvement in Nasal Symptoms of Chronic Rhinitis after Cryoablation of the Posterior Nasal Nerve

**DOI:** 10.1002/oto2.77

**Published:** 2023-10-17

**Authors:** Mattie Rosi‐Schumacher, Adam Abbas, Paul R. Young

**Affiliations:** ^1^ Department of Otolaryngology–Head and Neck Surgery, Jacobs School of Medicine and Biomedical Sciences, University at Buffalo State University of New York Buffalo New York USA; ^2^ Ear, Nose, and Throat, PLLC Buffalo New York USA

**Keywords:** allergic rhinitis, chronic rhinitis, cryoablation, cryotherapy, nonallergic rhinitis, posterior nasal nerve, total nasal symptom score

## Abstract

**Objective:**

To determine the efficacy of posterior nasal nerve (PNN) cryoablation for improving the symptoms of chronic rhinitis.

**Study Design:**

Retrospective cohort study.

**Setting:**

A private practice.

**Methods:**

This study evaluated medication usage and adverse effects of in‐office PNN cryoablation with a handheld device in patients > 18 years with chronic (>6 months) allergic or nonallergic rhinitis for whom medical management failed. The total nasal symptom score (TNSS) and mini rhinoconjunctivitis quality of life questionnaire (mRQLQ) scores were compared before and after treatment.

**Results:**

This study included 127 patients with a mean age of 52.4 ± 16.9 years; 60.6% of patients were female and 49.6% had allergic rhinitis. Mean symptom scores decreased from 5.94 (95% confidence interval [CI], 5.51‐6.43) to 3.44 (95% CI, 2.97‐3.81, *P* < .001) after the procedure, with clinically important decreases in 75 (59.1%) patients. For patients with baseline TNSS values of ≥4, 63.5% (66/104) had a clinically important decrease, whereas only 39.1% (9/23) of those with the lower baseline did (*P* = .04). Mean mRQLQ scores also decreased from 2.51 (95% CI, 2.29‐2.72) to 1.28 (95% CI, 1.20‐1.47, *P* < .001) after the procedure. Seventy‐eight of 273 (28.6%) medications were discontinued after the procedure. Adverse effects occurred in 18.1% (23/127) of patients with headache as the most common.

**Conclusion:**

PNN cryoablation improves nasal symptoms and quality of life in patients with chronic rhinitis. Patients with a higher baseline TNSS are more likely to experience significant symptomatic improvement.

Chronic rhinitis encompasses a variety of conditions involving nasal inflammation and/or dysfunction of the nasal mucosa and may present comorbid with asthma and conjunctivitis.^1^ The worldwide prevalence of chronic rhinitis is 29.4%, with allergic rhinitis (AR) and nonallergic rhinitis (NAR) affecting 18.1% and 12.0% of people, respectively.^2^ In the United States (U.S.), chronic rhinitis affects an estimated 400 million individuals.^3^, ^4^ Those with AR experience mucosal inflammation driven by type 2 helper T cells and immunoglobulin E (IgE)‐mediated reactions against inhaled allergens, resulting in sneezing, nasal pruritus, airflow obstruction, and clear nasal discharge.^5^ A positive result on a skin prick test or allergen‐specific IgE test is the determinant for a diagnosis of AR.^1^ NAR has an array of etiologies but presents as symptoms of rhinitis without positive allergy test results or evidence of infection.^1^

Chronic rhinitis can substantially impact an individual's quality of life and is associated with impaired sleep, negative psychological sequelae, and decreased productivity.^6^, ^7^, ^8^ The condition can also be a financial burden due to the costs of medical care, office visits, tests, medications, and missed work.^9^ The total direct medical cost of AR was estimated to be $3.4 billion in 2011 (which equates to ∼$4.6 billion in 2023 costs using the U.S. Bureau of Labor Statistics Inflation Calculator), with almost half of this cost attributable to prescription medications.^10^ Treatments of chronic rhinitis include intranasal steroids, antihistamines, and anticholinergics. However, these medications fail to ameliorate symptoms in 10% to 20% of patients who continue to experience postnasal drip, sneezing, and sleeping difficulties.^11^, ^12^ Nasal sprays can also produce unwanted side effects, such as a bitter taste and somnolence.^13^ The failure, in some instances, to relieve nasal symptoms can result in poor adherence to treatment.

Cases of chronic rhinitis that are refractory to medical management may be considered for procedural intervention. Vidian neurectomy is a procedure, first described in the 1960s by Golding‐Wood,^14^ that targets the preganglionic parasympathetic innervation to the secretory nasal mucosa.^15^ Some of the bothersome symptoms of rhinitis, including excessive rhinorrhea, are attributed to an imbalance of parasympathetic and sympathetic input.^16^, ^17^ The vidian nerve is transected to reduce nasal hypersecretion by eliminating most of the autonomic input to the nasal cavity.^16^, ^17^ Alternatively, endoscopic ablation of the posterior nasal nerve (PNN) can be performed to eliminate the postganglionic input to the nasal mucosa.^15^ This method has gained favor because it is less invasive and does not result in the denervation of the lacrimal gland.^16^

Cryoablation of the PNN can be performed in a health care provider's office. The U.S. Food and Drug Administration approved a handheld device (ClariFix; Stryker Corporation) for this purpose in 2017. This device uses nitrous oxide to freeze the mucosal tissue (to between −20°C and −100°C), resulting in second‐degree nerve damage.^18^ The efficacy of cryosurgery is due to its ability to destroy targeted cells and induce cell apoptosis and vascular stasis.^19^ This treatment reduces vascularity, mucosal thickness, and the size of glandular acini; reactive inflammation dissipates within 4 to 5 weeks.^20^ This study serves to (i) validate and extend previous findings on the efficacy of PNN cryoablation,^21^, ^22^, ^23^ (ii) to quantify the improvement in postnasal drip, and (iii) to determine the differential efficacy of the procedure according to baseline patient symptom scores.

## Methods

### Study Design and Patient Population

This was a retrospective study conducted at a private practice of a single otolaryngologist. Medical records of patients who sought treatment between April 2019 and September 2022 were reviewed. This study was reviewed and approved by the University at Buffalo Institutional Review Board.

Patients ≥18 years old with a diagnosis of chronic (>6 months) AR or NAR who consented to and received in‐office cryoablation of the PNN were selected for the study. Patients were determined to have AR via prior skin prick allergy testing or serum IgE‐antibody testing. Further inclusion criteria were (i) the presence of rhinorrhea and/or nasal congestion that was bothersome to the patient or interfered with normal life as detailed by the patient and physician and as documented in the electronic health record, and (ii) failure of medical management and inadequate symptom relief over at least 4 weeks of treatment with nasal medications, including steroids, antihistamines, and/or anticholinergics. Patients were not excluded based on medication use. Patients were excluded if they had any of the following: nasal or sinus infection, coagulation disorders, anticoagulant medication use, or anatomical obstruction in the nose. Patients were also excluded if they had incomplete survey data.

### Procedure

The PNN cryoablation procedure was performed in the office using a handheld cryotherapy device (ClariFix; Stryker Corporation). The intervention was performed under local anesthesia using topical anesthetic in the following manner: first, 6% ponticaine gel is applied topically just lateral to the middle turbinates for 5 minutes and then removed via suction, next, cotton pledgets soaked in a 50/50 mixture of 1:10,000 epinephrine and 4% lidocaine are placed lateral to the middle turbinates, and last, 1% lidocaine is injected submucosally into the root of the middle turbinates and area of the sphenopalatine foramen. The cryoablation device was prepared in the usual fashion with the nitrous oxide canister loaded into the cryoprobe. Under direct visualization using nasal endoscopy, the balloon tip of the cryodevice is inserted into the nose and applied with direct contact to the mucosa of the posterior middle meatus. Care is taken to ensure the balloon is in firm contact with the target tissue. The device is then activated using the valve on/off button for 30 seconds at 1 site. For optimum treatment, the cryoprobe is held stable in 1 location without movement of the balloon during cryoablation. To avoid unwanted tissue injury, the balloon is allowed to thaw prior to removal from the target tissue. The endoscope and handheld device are then passed into the contralateral nares and applied in an identical fashion. There were no other concurrent procedures performed at the time of the intervention. The device is designed for single use and is disposed of after the procedure.

### Data Collection

Data were manually retrieved from patient's electronic medical records between January to February 2023. Information related to the pretreatment visit, procedure visit, and posttreatment follow‐up was reviewed. Patient‐reported demographic information, including age, race, and sex, were collected. Information on the diagnoses of AR versus NAR, sinusitis, asthma, epistaxis, ear pain, migraine, and ocular symptoms was collected from the patient's medical histories. The dates of the preintervention survey, procedure, and postintervention survey were recorded. All relevant pre‐ and postprocedure medication use was recorded, including that for intranasal antihistamines (azelastine), intranasal steroids (fluticasone, beclomethasone, budesonide, and triamcinolone), intranasal anticholinergics (ipratropium), oral antihistamines (loratadine, fexofenadine, cetirizine, and cyproheptadine), oral antileukotrienes (montelukast), and oral expectorants (guaifenesin). Patients were not required to stop taking any medications prior to the procedure. During the posttreatment visit, information was recorded on adverse effects from the procedure, including facial pain and pressure, nasal dryness, epistaxis, headache, numbness of the palate, ocular symptoms, dizziness, or loss of smell/taste.

### Surveys

Patients were asked to complete 2 surveys before the procedure (baseline) and at a postprocedure follow‐up visit: the total nasal symptom score (TNSS) and the mini rhinoconjunctivitis quality of life questionnaire (mRQLQ). The TNSS asks patients to rate 4 categories of nasal symptoms (runny nose, nasal congestion, nasal itching, and sneezing) over the past week on a scale from 0 (no symptoms) to 3 (symptoms present and interfere with normal life). The total score was a sum of the values for each of the 4 items, with a possible range of 0 to 12. There was 1 free‐standing question on the pre‐ and posttreatment survey asking patients to rate their symptoms of postnasal drip on the same scale (from 0 to 3). There were no minimum requirements for pretreatment TNSS for inclusion in the study.

The mRQLQ includes 14 items to assess how a patient's daily life (regular or recreational activities at home and work) is affected by chronic symptoms, including the need to blow their nose repeatedly, sleep, need to rub nose/eyes, sneezing, stuffy/blocked nose, runny nose, itchy eyes, sore eyes, watery eyes, tiredness/fatigue, thirst, and feeling irritable. The patients were asked to rate how much their daily life was troubled by their rhinitis symptoms over the past week on a scale from 0 (not troubled) to 6 (extremely troubled). The score was calculated as an average of the 14 items, with a possible range from 0 to 6.

The primary endpoint of this study was the reduction in TNSS from the pretreatment baseline. The secondary endpoints were the improvement in mRQLQ scores from baseline and the rate of medication discontinuation.

### Data Analysis

Continuous data were summarized using descriptive statistics for normally distributed data (mean, 95% confidence interval [CI]). Categorical data were summarized as frequency counts and percentages. Statistical analyses were conducted using SPSS Statistics (version 28; IBM, Armonk). Survey score changes from baseline were analyzed using 2‐tailed paired *t* tests. The Wilcoxon signed‐rank test was used for data without a normal distribution. The effect size was described by Cohen's *d*, where a *d* of 0.5 represents a medium effect and *d* of ≥0.8 represents a large effect. Regression analysis was used to analyze the relation of multiple factors to the outcome. The minimal clinically important difference (MCID) has been established as a reduction of 30% in the TNSS, and was used to provide context to the findings of this study.^24^ A *P* value of .05 indicated statistical significance. A sample size calculation determined 96 subjects were required to have a confidence level of 95%.

## Results

One‐hundred thirty‐two patients received cryotherapy during the study period; 5 had incomplete survey data, leaving a total of 127 patients that were included in the study. The patients ranged from 20 to 90 years (mean, 52.4 years; 95% CI, 49.4‐55.3 years). Demographic information can be found in Table 1. Patients with NAR were significantly older than those with AR (58.2 [95% CI, 54.3‐62.0] years versus 46.5 [95% CI, 42.4‐50.5] years, respectively; *P* < .001). There were also significantly more females than males with AR (*P* = .001), but there were no other differences between those with AR and NAR. The timeframe for posttreatment follow‐up and survey collection ranged from 42 to 192 days. The mean follow‐up time was 51.8 days with a standard deviation of 18.8 days; the median was 46 days.

**Table 1:** Demographics of the Study Population

Category	No. (%)			*P* value^a^
	Total cohort (N = 127)	Non‐allergic rhinitis (N = 64, 50.4%)	Allergic rhinitis (N = 63, 49.6%)	
Sex				.001
Female	77 (60.6)	30 (46.9)	47 (74.6)	
Male	50 (39.4)	34 (53.1)	16 (25.4)	
Race				.368
White	118 (92.9)	58 (90.6)	60 (95.2)	
Black	5 (3.9)	4 (6.3)	1 (1.6)	
Asian	3 (2.4)	2 (3.1)	1 (1.6)	
Hispanic	1(0.8)	0 (0.0)	1 (1.6)	
Medical history^b^				
Sinusitis	47 (37.0)	22 (34.4)	25 (39.7)	.536
Asthma	9 (7.1)	2 (3.1)	7 (11.1)	.079
Epistaxis	2(1.6)	1 (1.6)	1 (1.6)	.982
Ear pain	11 (8.7)	4 (6.3)	7 (11.1)	.303
Migraines	13 (10.2)	4 (6.3)	9 (14.3)	.135
Ocular symptoms	5 (3.9)	3 (4.7)	2 (3.2)	.661

^a^ Pearson chi‐square test between nonallergic and allergic rhinitis.

^b^ Values represent counts of the condition, not number of patients, as some patients had multiple comorbidities.

### Nasal Symptom Survey Results

**Figure 1:**
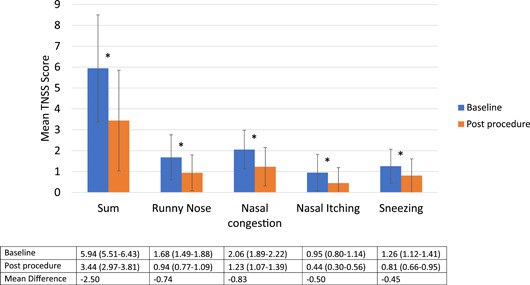
Total nasal symptom score (TNSS) values for the total study population. Error bars mark the standard deviation. Values in the chart are: mean (95% confidence interval). *P < .001 by paired samples *t* test.

Overall, for the combined cohort of AR and NAR patients, TNSS values and scores on each of the 4 items decreased significantly after PNN ablation (Figure 1); Cohen's *d* = 0.94 for the difference between the sum values. An MCID was attained by 75 (59.1%) patients. The greatest improvement among the TNSS domains was for nasal congestion (average difference of −0.83 from baseline; Cohen's *d* = 0.78) followed by runny nose (difference, −0.74, Cohen's *d* = 0.78). Furthermore, patient‐rated postnasal drip decreased a mean of 1.09 points in the total cohort, from 2.64 (95% CI, 2.58‐2.69) at baseline to 1.55 (95% CI, 1.46‐1.63; *P* < .001) postprocedure (Cohen's *d* = 1.11).

### NAR Versus AR

The degree of improvement in TNSS over baseline was statistically comparable between patients with AR and NAR (Table 2; *P* = .085). Patients with AR had higher preoperative ratings of nasal itching (*P* = .006), and experienced a greater magnitude of improvement in this symptom as well (*P* = .012). The end‐point ratings of nasal itching after the procedure were not significantly different between AR and NAR patients. Patients with AR and NAR did not differ significantly in the categories of runny nose, nasal congestion, sneezing, or postnasal drip.

**Table 2:** Nonallergic (NAR) versus Allergic (AR) Rhinitis Nasal Symptom Results

	NAR baseline		AR baseline			NAR post		AR post			NAR		AR		
Metric	Mean	SD	Mean	SD	*P* value	Mean	SD	Mean	SD	*P* value	Difference	SD	Difference	SD	*P* value
TNSS sum	5.71	2.81	6.44	2.45	.117	3.49	2.65	3.51	2.37	.972	−2.22	3.15	−2.94	2.73	.085
Runny nose	1.69	1.17	1.70	0.98	.955	1.00	0.88	0.89	0.87	.470	−0.67	1.08	−0.82	1.08	.218
Nasal congestion	2.03	1.02	2.11	0.81	.621	1.30	0.94	1.29	0.94	.947	−0.72	1.13	−0.83	1.01	.288
Nasal itching	0.78	0.79	1.17	0.94	.006*	0.43	0.77	0.48	0.76	.348	−0.38	0.79	−0.71	0.86	.012*
Sneezing	1.17	0.81	1.37	0.83	.098	0.78	0.84	0.86	0.80	.309	−0.41	0.78	−0.51	0.84	.255
Postnasal drip	2.63	0.64	2.65	0.58	.838	1.68	0.92	1.40	0.96	.094	−0.97	0.98	−1.22	0.99	.085

Abbreviation: TNSS, total nasal symptom score.

* Represents statistically significant values.

### Analysis Based on Preoperative Symptom Rating

There were 104 patients who had a baseline TNSS of ≥4. In this subcohort, the mean baseline score was 6.82 (95% CI, 6.45‐7.20) and the postprocedure score was 3.48 (95% CI, 3.00‐3.96; *P* < .001; Cohen's *d* = 1.24). For the remaining 23 patients, the low baseline TNSS (2.09 [95% CI, 1.66‐2.52]) did not differ significantly from the postprocedure score (2.23 [95% CI, 1.44‐3.01], *P* = .070). Furthermore, 63.5% (66/104) of patients with baseline TNSS values of ≥4 had an MCID, whereas only 39.1% (9/23) of those with the lower baseline did (*P* = .04).

### Quality of Life Survey Results

**Figure 2:**
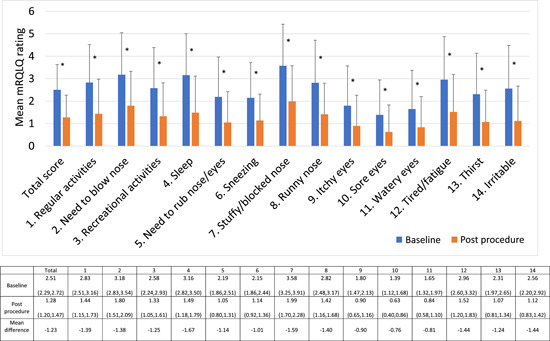
Mini Rhinoconjunctivitis Quality of Life Questionnaire (mRQLQ) scores for the total study population. Error bars mark the standard deviation. Values in the chart are: mean (95% confidence Interval). *P < .001 by Wilcoxon signed‐rank test.

Among all patients, the overall mRQLQ score and scores for each of the items decreased significantly after cryoablation (Figure 2); Cohen's *d* = 1.29 for the difference between the total scores. The decreases were similar between those with AR and those with NAR (*P* = .299). The greatest improvement was seen for sleep (difference of −1.67 from baseline) and stuffy/blocked nose (difference, −1.59).

A regression analysis showed that neither demographic factors (age, race, sex, and past medical history) nor preoperative ratings on TNSS had a significant effect on the outcome scores (TNSS or mRQLQ).

### Medication Use and Adverse Effects

The medications used before and discontinued after the cryoablation procedure are listed in Table 3. Although intranasal corticosteroids and antihistamines were the most commonly used, oral expectorants were the most likely to be discontinued. Adverse effects were reported by 18.1% (23/127) of patients, with headaches reported most frequently (Table 4). However, patients reported that headaches, facial pain, and dizziness were transient, only lasting hours to days after the procedure. The incidents of epistaxis were mild and self‐limited and did not require nasal packing or operative intervention. On further follow‐up, patients reported resolution of hyposmia/dysgeusia and roof‐of‐mouth hypoesthesia by 12 weeks postprocedure.

**Table 3:** Medication Use

Class	Number at baseline	Number (%) discontinued
Corticosteroid (intranasal)	102	23 (22.5)
Antihistamine (intranasal)	82	22 (26.8)
Antihistamine (oral)	45	8 (17.8)
Anticholinergic (intranasal)	32	14 (43.8)
Antileukotriene (oral)	12	5 (41.7)
Expectorant (oral)	10	6 (60.0)
Total	273	78 (28.6)

**Table 4:** Adverse Effects^a^

Description	Number (%) of patients
Headache	8 (6.3)
Mild facial pain and pressure	6 (4.7)
Epistaxis^b^	4 (3.1)
Nasal dryness	2 (1.6)
Dizziness	2 (1.6)
Hyposmia or dysgeusia	2 (1.6)
Roof‐of‐mouth hypoesthesia	1 (0.8)
Ocular symptoms (dry or watery eyes)	0 (0.0)

^a^ A total of 22 patients experienced 1 side effect; 1 patient reported 3 (mild facial pain, epistaxis, and headache).

^b^ One patient had a past medical history of epistaxis.

## Discussion

We describe the outcomes of cryotherapy with a handheld device for the treatment of chronic rhinitis in a large cohort of patients. This study assessed the most inclusive and generalizable patient cohort among those in current published literature, without a minimum TNSS value used as a prerequisite for study inclusion and no patient exclusion based on preoperative medication usage. We found that a higher baseline TNSS was associated with greater improvement at follow‐up, and a greater proportion of patients with high TNSS values attained the MCID.

Our study further validates previous findings that cryoablation provides clinical benefits to patients with both NAR and AR.^21^, ^22^ Prior surgical outcome studies have demonstrated that patients with AR who underwent vidian neurectomy or selective endoscopic PNN neurectomy experienced an improvement in nasal symptoms of nasal obstruction and rhinorrhea and a decrease in mean TNSS score.^25^, ^26^ Neurectomy denervates the nasal mucosa and is hypothesized to prevent or dampen downstream allergic reactions by rendering the nasal tissue unresponsive to allergens. A microscopic analysis of biopsy specimens from nasal mucosa following PNN resection found a significant reduction in the number of inflammatory cells including neutrophils, eosinophils, and lymphocytes.^27^ Therefore, it is suspected that disruption of the autonomic innervation leads to a reduction in the pathologic inflammatory reactions of both AR and NAR. Our study demonstrated similar improvements across all nasal symptoms for both AR and NAR patients, with a more significant improvement in nasal itching for patients with AR. Notably, while patients were assigned to 1 subcohort based on allergy testing, patients with a mixed rhinitis subtype may be difficult to characterize and experience improvement from predominantly non‐allergic symptoms.

The first report on office‐based cryotherapy for the treatment of chronic rhinitis was published by Hwang et al^21^ in 2017 and described a prospective study with 27 patients at 3 centers. The patient population in that study was similar to ours, as the mean age was 53.3 ± 3.3 years, 63% were female, and 48% had AR. However, only patients with a baseline TNSS for rhinorrhea and/or congestion of ≥2 were included, which may explain the greater absolute reduction of TNSS scores in that study: baseline TNSS was 6.2 ± 0.5, which decreased to 2.6 ± 0.3 at 30 days (absolute reduction of 3.6). Hwang et al^21^ collected postintervention data at 5 time points: 7, 30, 90, 180, and 365 days after the procedure. They observed similar/stable reductions in scores at all time points, though they ultimately lost 18 patients to follow‐up. Similar to our findings, only 25.9% (7/27) of patients in their study reported adverse effects or mild pain/discomfort on the first day: overall, twelve patients reported ear blockage, 2 reported nasal dryness, and 1 reported a nosebleed. Unlike our study, the mRQLQ or medication use was not evaluated.

In 2019, Chang et al^22^ reported a prospective trial of 98 patients from 6 centers who underwent office‐based cryotherapy for the treatment of chronic rhinitis. The patient population in their study was of a similar mean age and gender distribution (58.6 ± 16.2 years and 64.3% female, respectively) as that in our study but only 28.6% had AR. Their analysis included multiple time points (30, 90, 180, and 270 days after the procedure) and a minimum TNSS of 4 was required for inclusion as well as a TNSS rating of 2 or 3 for rhinorrhea and 1 to 3 for congestion. Furthermore, participants were required to discontinue use of nasal anticholinergics at least 3 days before and throughout study participation. Therefore, their study only represented participants with severe baseline disease symptomatology, which may explain the substantial benefit perceived from the procedure: improvement from a baseline TNSS of 6.1 ± 1.9 to 2.9 ± 1.9 at 30 days (absolute reduction of 3.2).^22^ The ratings remained stable at later time points, suggesting that the maximal degree of improvement was captured at the 30‐day time point even though 5 patients were lost to follow‐up over time. More than 3 quarters of their patients (76/97 [78.4%]) had an MCID at 30 days, which was higher than what we observed (75/127 [59.1%]). However, when we analyzed patients with a baseline TNSS of ≥4, the proportion of patients that had an MCID increased to 63.5%. Chang et al also used the RQLQ (28‐item questionnaire) and found that the scores decreased from 3.0 ± 1.0 to 1.5 ± 1.2 at 90 days, which was comparable to our study.^22^ They similarly reported 29 adverse events, including epistaxis, facial pain, headache, dry eyes, bad taste, and oral numbness. In addition, 33/154 (21.4%) medications were discontinued during the study period, which was similar to the proportion we found in our study. Ow et al^23^ extended this trial to assess long‐term (12‐24 months) results. From the 91 patients followed up at 12 months after the procedure, the TNSS value was 3.0 points lower than baseline (median, −3; interquartile range, −4.0 to −1.0).^23^ After 24 months, the median improvement in the TNSS value from 57 patients was −4.0 (interquartile range, −5.0 to −2.0): 80.7% of patients had met the criteria for the MCID in TNSS at 24 months. Similarly, the median improvement in the RQLQ was −2.1 (interquartile range, −3.0 to −0.8) at 24 months, with 77.2% of patients achieving the MCID. This study demonstrates the long‐term efficacy of PNN cryoablation.

This is the first study to provide evidence for the differential clinical efficacy of PNN cryoablation based on preoperative symptom scores. This may provide physicians with an indication to seek alternative treatment modalities for those patients not meeting the threshold on preoperative nasal symptom screening. This is also the first study to quantify data on patient‐rated symptoms of postnasal drip after treatment with this procedure, which demonstrated the greatest decrease and symptomatic improvement across all measured nasal subdomains. This is also reflected in the fact that the majority of patients using expectorants preoperatively discontinued these medications after the procedure. This information may allow physicians to gauge the likelihood of an individual patient experiencing symptom improvement and to set realistic patient expectations for the utility of the procedure and likely clinical outcomes during the preoperative discussion.

A limitation of this study is that the analyses were retrospective. In addition, data regarding adverse events were subject to possible recall bias, because patients were asked to report complications retrospectively at their follow‐up appointment. While the major weakness of this study is the short follow‐up duration, prior studies have already demonstrated long‐lasting results up to 2 years after the cryoablation procedure as discussed above.^22^, ^23^ Further, histological studies by Kellerhals et al have shown that the end results of cryotherapy in the nasal mucosa (reduced vascularity, mucosal thickness, and size of glandular acini) are achieved at 4 to 5 weeks after the procedure, a time point which is captured in our study.^20^ Previously published data reported by Hwang et al^21^ and Chang et al^22^ at 30 days postprocedure did not demonstrate significant further improvement at later time points, suggesting our data likely portrays the clinically significant endpoint.^21^, ^22^

## Conclusion

In‐office cryoablation of the PNN results in significant improvement in patient‐rated nasal symptom scores and quality of life. The largest symptom improvement was seen with postnasal drip and was comparable in patients with AR and NAR. Patients with a higher baseline TNSS are more likely to experience significant symptomatic improvement.

## Author Contributions

**Mattie Rosi‐Schumacher**, conceptualization and design of the work, data acquisition, analysis, and interpretation, writing—original draft and revision of the manuscript, final approval of the manuscript, accountable for appropriate portions of the content; **Adam Abbas**, conceptualization and design of the work, data acquisition, analysis, and interpretation, writing—original draft and revision of the manuscript, approved final version of the manuscript, accountable for appropriate portions of the content; **Paul R. Young**, conceptualization and design of the work, data acquisition, analysis, and interpretation, writing—original draft and revision of the manuscript, approved final version of the manuscript, accountable for appropriate portions of the content.

## Disclosures

### Competing interests

The authors declare that there is no conflict of interest.

### Funding source

No funding was received for this work.
